# Dairy Cattle and the Iconic Autochthonous Cattle in Northern Portugal Are Reservoirs of Multidrug-Resistant *Escherichia coli*

**DOI:** 10.3390/antibiotics13121208

**Published:** 2024-12-11

**Authors:** Sandra Quinteira, Rui Dantas, Luís Pinho, Carla Campos, Ana R. Freitas, Nuno V. Brito, Carla Miranda

**Affiliations:** 1 CIBIO—Research Center in Biodiversity and Genetic Resources, InBIO, Research Network in Biodiversity and Evolutionary Biology, Associated Laboratory, University of Porto, Campus de Vairão, Rua Padre Armando Quintas 7, 4485-661 Vairão, Portugal; sandra.quinteira@ipsn.cespu.pt; 2 BIOPOLIS Program in Genomics, Biodiversity and Land Planning, Campus de Vairão, Rua Padre Armando Quintas 7, 4485-661 Vairão, Portugal; 3 Department of Biology, Faculty of Sciences, University of Porto, Rua do Campo Alegre s/n, 4169-007 Porto, Portugal; 4 UCIBIO—Applied Molecular Biosciences Unit, University Institute of Health Sciences (1H-TOXRUN, IUCS-CESPU), Avenida Central de Gandra 1317, 4585-116 Paredes, Portugal; rui.dantas@iucs.cespu.pt (R.D.); ana.freitas@iucs.cespu.pt (A.R.F.); nuno.brito@iucs.cespu.pt (N.V.B.); 5 ACRC—Associação Criadores da Raça Cachena, Parque Empresarial de Paçô, Rua da Roca 107, 4970-249 Arcos de Valdevez, Portugal; 6 Department of Veterinary Clinics, Abel Salazar Biomedical Sciences Institute, University of Porto, Rua Jorge de Viterbo Ferreira 228, 4050-313 Porto, Portugal; lapinho@icbas.up.pt; 7 Instituto Português de Oncologia do Porto Francisco Gentil, Rua Dr. António Bernardino de Almeida, 4200-072 Porto, Portugal; carla.campos@ipoporto.min-saude.pt; 8 Escola Superior de Saúde, Instituto Politécnico do Porto, Rua Dr. António Bernardino de Almeida, 4200-072 Porto, Portugal; 9 UCIBIO—Applied Molecular Biosciences Unit, Faculty of Pharmacy, University of Porto, Rua Jorge de Viterbo Ferreira 228, 4050-313 Porto, Portugal; 10 Associate Laboratory i4HB, Institute for Health and Bioeconomy, Faculty of Pharmacy, University of Porto, Rua Jorge de Viterbo Ferreira 228, 4050-313 Porto, Portugal; 11 CISAS—Center for Research and Development in Agrifood Systems and Sustainability, Higher Agricultural School, Polytechnic Institute of Viana do Castelo, Rua Escola Industrial e Comercial de Nun’Álvares, 4900-347 Viana do Castelo, Portugal; 12 LAQV-REQUIMTE—Associated Laboratory for Green Chemistry of the Network of Chemistry and Technology, University NOVA of Lisbon, Campus da Caparica, 1099-085 Caparica, Portugal

**Keywords:** antimicrobial resistance, *Escherichia coli*, livestock, One Health, native breeds

## Abstract

**Background/Objectives:** Animals destined for human consumption play a key role in potentially transmitting bacteria carrying antibiotic resistance genes. However, there is limited knowledge about the carriage of antibiotic-resistant bacteria in native breeds. We aimed to characterize the phenotypic profiles and antibiotic resistance genes in *Escherichia coli* isolated from bovines, including three native Portuguese bovine breeds. **Methods:** Forty-nine *E. coli* isolates were selected from 640 fecal samples pooled by age group (eight adult or eight calf samples) from each farm, representing both dairy cattle raised in intensive systems and meat cattle raised in extensive systems in Northern Portugal. The presumptive *E. coli* colonies plated onto MacConkey agar were confirmed using matrix-assisted laser desorption ionization time-of-flight mass spectrometry (MALDI-TOF MS). The antibiotic resistance profiles were screened by antimicrobial susceptibility testing (EUCAST/CLSI guidelines), and the antibiotic resistance genes by PCR. **Results:** Most isolates showed resistance to ampicillin (69%), tetracycline (57%), gentamicin (55%), and trimethoprim + sulfamethoxazole (53%), with no resistance to imipenem. Resistance to at least one antibiotic was found in 92% of isolates, while 59% exhibited multidrug resistance. Most calf isolates, including those from native breeds, showed a multidrug-resistant phenotype. Among the adults, this was only observed in Holstein-Friesian and Barrosã cattle. None of the Holstein-Friesian isolates were susceptible to all the tested antibiotics. ESBL-producing *E. coli* was identified in 39% of isolates, including those from Holstein-Friesian calves and adults, Cachena calves and Minhota adults. The *sul*2 gene was detected in 69% of isolates, followed by *bla*_CTX-M_ (45%), *aac*(3′)-IV (41%), and *aac*(6′)-Ib-cr (31%), with a higher prevalence in adults. **Conclusions:** This pioneering study highlights the concerning presence of multidrug-resistant *E. coli* in native Portuguese cattle breeds.

## 1. Introduction

Antimicrobial resistance (AMR) is among the top ten threats to global public health, requiring multidisciplinary and intersectoral actions, such as the One Health approach, to address what is increasingly referred to as a “silent pandemic” [1]. It is also essential to reduce the 10 million annual deaths projected to be attributable to AMR by 2050 [1]. This crisis has been exacerbated by the inappropriate use and overuse of antibiotics, which have contributed to the acceleration of natural selection and the emergence of antibiotic-resistant bacteria through acquired mutations and antimicrobial resistance genes [2,3]. The Quadripartite Alliance, comprising the Food and Agriculture Organization of the United Nations (FAO), the World Health Organization (WHO), the UN Environment Program (UNEP), and the World Organization for Animal Health (WOAH), has recently emerged to combat AMR and promote the prudent use of antimicrobials in human and veterinary medicine, as well as in agriculture [4].

The use of antimicrobials in food-producing animals has shown a downward trend since 2014. Between 2014 and 2021, the total antimicrobial use in these animals decreased by 44%, while human consumption remained stable. Global antimicrobial use in animals specifically decreased by 13% between 2017 and 2019 [5,6]. However, the most recent WOAH report indicated a 2% increase in antimicrobial use in 2021. This increase could be attributed to a slowdown in the global reduction of antimicrobial usage and improvements in reporting accuracy, despite a significant reduction in the use of antimicrobials for growth promotion [7]. Tetracyclines, followed by penicillins, were the most widely used antimicrobials in animal health worldwide in 2021 [7,8].

Animals can act as reservoirs of antibiotic-resistant bacteria, such as those belonging to the order Enterobacteriales, including *Escherichia coli*, which may exhibit resistance to carbapenems and/or produce extended-spectrum beta-lactamases (ESBLs, enzymes produced by some bacteria that can break down and inactivate a wide range of beta-lactam antibiotics like penicillins and cephalosporins), making infections harder to treat. These bacteria are included in the WHO’s global priority list of pathogens for which there are limited therapeutic options [9,10]. Currently, *E. coli*, including both pathogenic strains and commensals (which can become pathogenic in immunosuppressed hosts), is a leading cause of deadly infections, including pneumonia and bloodstream infections associated with multidrug-resistant (MDR, resistant to ≥3 antimicrobial classes) phenotypes. *Escherichia coli* can spread from food-producing animals to humans through the food supply, direct contact, or environmental exposure [2,8,9,11]. The One Health concept has been applied to AMR, focusing on the epidemiology and monitoring of multidrug-resistant microorganisms by integrating human, animal, and environmental health [10]. 

The analysis of the AMR profiles of *E. coli* from dairy farming in Portugal is limited [12], and data on antibiotic-resistant bacteria from native cattle breeds, which are raised in production systems with supposedly low antibiotic use, are currently nonexistent. Native Portuguese breeds represent a unique genetic heritage, adapted to the country’s diverse soil and climate conditions. The 15 indigenous cattle breeds in Portugal, some of which are at urgent risk of extinction, are primarily raised in extensive production systems. These systems contribute to environmental and landscape preservation by utilizing scarce mountain forage resources or underutilized disadvantaged areas. They also provide traditional, high-quality, safe products of significant economic value, often with Protected Designation of Origin [13,14]. These breeds play a fundamental role in maintaining the social, historical-cultural, and ecological balance, and they can enhance the profitability of agricultural holdings, support population settlement, and stimulate the regional economy. Although the administration of antimicrobials in these breeds intended for human consumption is limited—and in some, even absent—the available data on the application of these compounds and the prevalence of antimicrobial resistance in clinically relevant bacteria remain scarce or even nonexistent [13,14]. Aligned with a One Health approach, this study focused on characterizing the phenotypic profiles and antibiotic resistance genes in *E. coli* isolated from bovines, including three native Portuguese bovine breeds that often inhabit inhospitable locations.

## 2. Results

### 2.1. Selection of Isolates for Study

All the pooled samples from both age groups—calves and cows—showed characteristic *E. coli* colonies, except for two adult pools, one from the Barrosã breed and another from the Cachena breed. A total of 347 isolates displaying typical *E. coli* morphology were collected, with 152 isolates obtained from the Holstein-Friesian breed and the remaining 195 from native breeds. Of these, 130 *E. coli* isolates were identified using matrix-assisted laser desorption ionization time-of-flight mass spectrometry (MALDI-TOF MS).

Based on criteria including representative breeds, animal age groups (calves/adults) and farm origin, 49 isolates from 21 farms were selected. These isolates were obtained from the pooled fecal samples of cattle from both dairy-intensive production (Holstein-Friesian breed: 13 calves and 16 adults) and extensive production with native Portuguese breeds (Barrosã: 6 calves and 6 adults; Cachena: 3 calves and 2 adults; Minhota: 2 calves and 1 adult).

### 2.2. Antimicrobial Susceptibility

**Figure 1:**
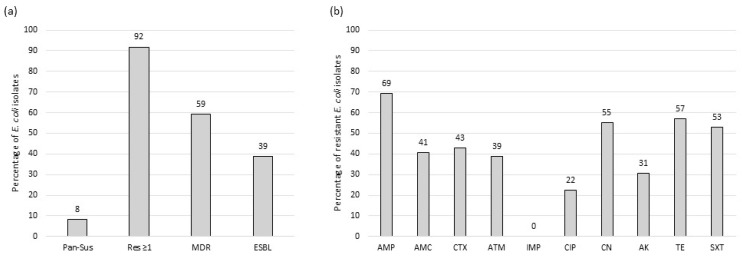
Phenotypic characterization of antimicrobial resistance in *E. coli* from cattle breeds (n = 49): (**a**) antimicrobial susceptibility profiles; and (**b**) antimicrobial resistance of *E. coli* isolates. Pan-sus—pan-susceptibility; Res ≥1—resistance to ≥1 antibiotic; MDR—multidrug-resistant phenotype; ESBL—extended-spectrum β-lactamase production phenotype; AMP: ampicillin; AMC: amoxicillin + clavulanic acid; CTX: cefotaxime; ATM: aztreonam; IPM: imipenem; CIP: ciprofloxacin; CN: gentamicin; AK: amikacin; TE: tetracycline; SXT: trimethoprim + sulfamethoxazole.

Most of the isolates exhibited antibiotic resistance to ampicillin (69%), tetracycline (57%), gentamicin (55%), and trimethoprim + sulfamethoxazole (53%). This was followed by resistance to cefotaxime (43%), amoxicillin + clavulanic acid (41%), aztreonam (39%), amikacin (31%), and ciprofloxacin (22%). None of the isolates showed resistance to imipenem. A total of 45 isolates (92%) were resistant to at least one antibiotic, while 29 isolates (59%) exhibited resistance to three or more antimicrobial classes, indicating a multidrug-resistant profile. The remaining four isolates (8%) were not resistant to any of the antibiotics tested (Figure 1 and Figure S1).

**Figure 2:**
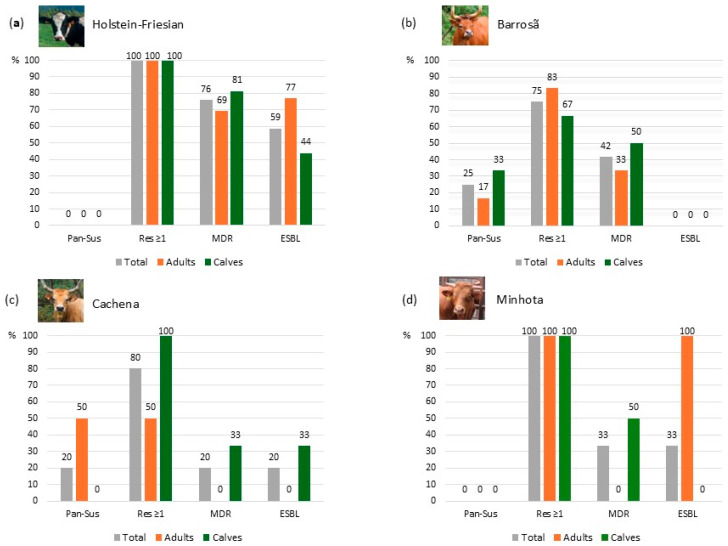
Antimicrobial susceptibility profiles of *E. coli* isolates from cattle breeds: (**a**) Holstein-Friesian breed (n = 29, with 16 from adults and 13 from calves); (**b**) Barrosã breed (n = 12, with 6 from adults and 6 from calves); (**c**) Cachena breed (n = 5, with 2 from adults and 3 from calves); and (**d**) Minhota breed (n = 3, with 1 from an adult and 2 from calves). Pan-sus: pan-susceptibility; Res ≥ 1: resistance to ≥1 antibiotics; MDR: multidrug-resistant phenotype; ESBL: extended-spectrum β-lactamase production phenotype.

Among the phenotypic antibiotic resistance profiles of the isolates obtained per breed, all the isolates from calves and adults of the Holstein-Friesian and Minhota breeds, as well as Cachena calves, were resistant to at least one antibiotic (Figure 2). In all the breeds, including the native Portuguese breeds, the majority of isolates from calves demonstrated a multidrug-resistant phenotype. For the isolates from adults, this was only observed in Holstein-Friesian and Barrosã cattle. None of the isolates from Holstein-Friesian cattle were susceptible to all the tested antibiotics. Of the nine Holstein-Friesian farms, all the *E. coli* isolates from both calves and adults in five farms exhibited an MDR profile. In two farms, only isolates from calves showed an MDR profile, while three isolates from a single farm did not exhibit an MDR profile (Table S1). Among the seven Barrosã farms, only isolates from calves were multidrug-resistant in two farms, and only isolates from adults were multidrug-resistant in two other farms, while all the isolates from calves and adults in two farms were not multidrug-resistant. From the three Cachena farms, the only isolate with an MDR phenotype came from a calf, and from the two Minhota farms, only the *E. coli* isolate from a calf was multidrug-resistant, from the farm with isolates from both calves and adults.

ESBL production was exhibited in 19 (39%) *E. coli* isolates from fecal samples of cattle (Figure 1). ESBL-producing *E. coli* isolates were found in both calves and adults of the Holstein-Friesian breed, as well as in Cachena calves and Minhota adults. In contrast, no ESBL activity was detected in the isolates from Barrosã cattle (Figure 2).

**Figure 3:**
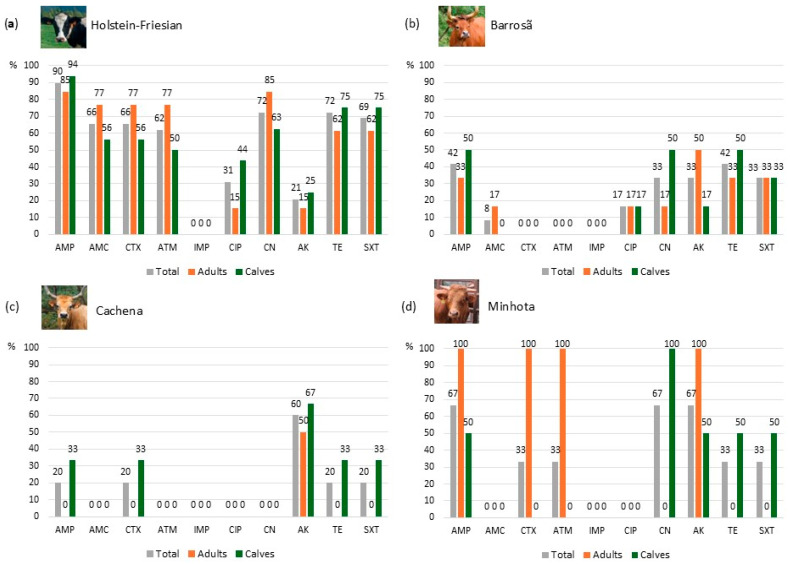
Antibiotic susceptibility of *E. coli* isolates from cattle breeds: (**a**) Holstein-Friesian breed (n = 29, with 16 from adults and 13 from calves); (**b**) Barrosã breed (n = 12, with 6 from adults and 6 from calves); (**c**) Cachena breed (n = 5, with 2 from adults and 3 from calves); and (**d**) Minhota breed (n = 3, with 1 from an adult and 2 from calves). AMP: ampicillin; AMC: amoxicillin + clavulanic acid; CTX: cefotaxime; ATM: aztreonam; IPM: imipenem; CIP: ciprofloxacin; CN: gentamicin; AK: amikacin; TE: tetracycline; SXT: trimethoprim + sulfamethoxazole.

For each cattle breed, the antibiotic resistance profile of the *E. coli* isolates is shown in Figure 3. With the exception of the resistance observed to amikacin and ciprofloxacin, the majority of isolates from dairy cattle raised in intensive systems exhibited high resistance to the remaining antibiotics. Resistance to ampicillin, tetracycline and trimethoprim + sulfamethoxazole was more frequent in isolates from calves than in those from adults (Figure 3a). Isolates from cattle raised in extensive farming systems generally exhibited lower levels of resistance. None of the isolates from any breed showed resistance to imipenem, as previously reported. Moreover, all the isolates from Barrosã cattle were susceptible to cefotaxime and aztreonam, while the isolates from Cachena cattle were susceptible to amoxicillin + clavulanic acid, aztreonam, ciprofloxacin, and gentamicin (Figure 3b,c). The isolates from Minhota cattle were susceptible to amoxicillin + clavulanic acid and ciprofloxacin (Figure 3d). Additionally, some antibiotic resistance was present only in one age group (adults or calves), as observed in Cachena calves for most antibiotics (Figure 3c).

### 2.3. Molecular Epidemiology

**Figure 4:**
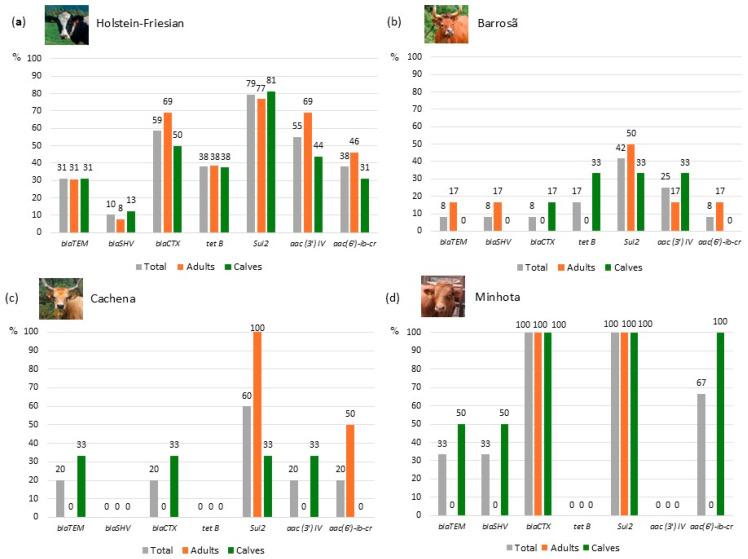
Genotypic characterization of the antimicrobial resistance profile of the *E. coli* (n = 49) isolated from all the cattle breeds: (**a**) Holstein-Friesian (n = 29, with 16 from adults and 13 from calves); (**b**) Barrosã (n = 12, with 6 from adults and 6 from calves); (**c**) Cachena (n = 5, with 2 from adults and 3 from calves); and (**d**) Minhota (n = 3, with 1 from an adult and 2 from calves).

All the tested genes encoding resistance to different classes of antibiotics were detected in this study (Table 1, Figure S2), particularly in the *E. coli* isolates obtained from Holstein-Friesian cattle (Figure 4). The majority (69%) of *E. coli* isolates harbored the *sul*2 gene, followed by *bla*_CTX-M_, *aac*(3′)-IV and *aac*(6′)-Ib-cr. These antibiotic resistance genes (*bla*_CTX-M_, *sul*2, *aac*(3′)-IV, and *aac*(6′)-Ib-cr) were more frequent in isolates from adults than in those from calves. Conversely, antibiotic resistance genes such as *bla*_TEM_, *bla*_SHV,_ and *tet*B were more frequent in isolates from calves than in those from adults (Table 1).

The amplification of beta-lactam resistance genes (*bla*_TEM_, *bla*_SHV_ and *bla*_CTX-M_) varied among the isolates, with several *E. coli* isolates, mostly from Holstein-Friesian cattle, harboring two or three of these genes (Table S1). No *E. coli* isolates from Cachena cattle harbored *bla*_SHV,_ although only isolates from calves amplified *bla*_TEM_ and *bla*_CTX-M_. The *bla*_TEM_ and *bla*_SHV_ genes were detected in isolates from Minhota calves and Barrosã adults. The presence of *bla*_CTX-M_ was observed in isolates from both animal age groups of the Minhota breed, while in the Barrosã breed, it was only detected in isolates from calves (Figure 4). The *tet*B and *aac*(3′)-IV genes were not amplified, possibly due to the small number of tested isolates, as was the case with *tet*B in Cachena and Minhota isolates. However, 55% and 38% of isolates from the Holstein-Friesian breed showed *aac*(3′)-IV and *tet*B genes, respectively (Figure 4 and Figure S2).

As expected, the isolates from the native cattle breeds showed fewer resistance genes than those from the Holstein-Friesian breed. Half of the tested isolates from Barrosã and Cachena cattle did not harbor antibiotic resistance genes, with six and two isolates, respectively. In contrast, only 3 out of 29 isolates obtained from Holstein-Friesian cattle did not show the presence of any of the tested genes (Table S1).

## 3. Discussion

Our pioneering study demonstrated the occurrence of MDR *E. coli* in cattle intended for human consumption, based on fecal samples collected from four distinct breeds, one of them raised in an intensive system or designed for dairy cattle and three native Portuguese cattle breeds raised in extensive production systems with limited antibiotic use. The *Escherichia coli* isolates from Holstein-Friesian cattle (including calves and adults) exhibited a broader antibiotic resistance profile compared to those from the other breeds, like Barrosã, Cachena and Minhota (Figure S1). These results may reflect the more frequent use of antibiotics in the intensive production system, which was confirmed in the three months preceding sample collection. Cephalosporins and penicillins—both part of the beta-lactam antibiotic class—are reported to be the most frequently used antibiotics for treating mastitis and other diseases in dairy cattle, including in Portugal [15]. 

*Escherichia coli* is becoming a significant global health threat due to its rapid spread and multidrug-resistant phenotypes present in humans, animals and the environment [15]. The dairy farm environment could be a source of pathogens causing mastitis in dairy cattle and could facilitate the horizontal transfer of antibiotic resistance and virulence genes from bacterial strains like *E. coli* to humans through contaminated meat and milk, agricultural crops, and surface water [16]. Sohaib et al. [16] isolated *E. coli* strains from a dairy environment in China, including blank soil (0.3%), crop field soil (0.6%), raw milk (25%), fecal samples (35%), and manure slurry (39%). In a study of a dairy cattle farm in Poland, two multidrug-resistant *E. coli* strains obtained from manure showed resistance to beta-lactams, aminoglycosides, tetracyclines, trimethoprim + sulfamethoxazole, and fluoroquinolones [17]. However, limited information is available regarding the antibiotic usage, the prevalence, and the antibiotic resistance profiles of this species in the native cattle breeds, particularly on Portuguese farms, making it difficult to compare the results obtained in the present study.

Resistance to at least one antibiotic was found in 92% of our isolates, while 59% exhibited multidrug resistance. When considering only the antimicrobial resistance profile of *E. coli* isolated from Holstein-Friesian cattle, the results showed that 100% were resistant to at least one antibiotic (Figure S1), consistent with previous findings [16], which was not the case for the isolates from native breeds (80%). Regarding the animal age, most isolates from calves, including those from native breeds, showed a multidrug-resistant phenotype. Among adult cattle, this was only observed in the Holstein-Friesian and Barrosã breeds. None of the Holstein-Friesian isolates were susceptible to all the tested antibiotics. Most isolates showed resistance to ampicillin (69%), tetracycline (57%), gentamicin (55%) and trimethoprim + sulfamethoxazole (53%). Similar patterns of resistance have been reported, with most fecal *E. coli* isolates exhibiting higher resistance to trimethoprim + sulfamethoxazole, cefotaxime, ampicillin, followed by ciprofloxacin and tetracycline [16]. In another study in South Korea, 48% of the *E. coli* isolated from the feces of healthy cattle between 2010 and 2020 were resistant to at least one antimicrobial agent, with only 17% representing a multidrug-resistant profile. Fewer than half of the isolates showed antibiotic resistance to tetracycline (41%) and streptomycin (39%), while resistance to the remaining tested antibiotics was ≤12% [18]. Notably, ampicillin, amoxicillin-clavulanic acid, tetracycline, and trimethoprim + sulfamethoxazole are considered highly important antibiotics, while gentamicin and amikacin are classified as critically important, and ciprofloxacin and cefotaxime are classified as highest priority critically important antimicrobials by the WHO. However, imipenem and aztreonam are used exclusively in humans [8]. All the isolates were susceptible to imipenem, an antibiotic restricted to hospital use, including those from Holstein-Friesian cattle. Consistently, all the isolates were also susceptible to meropenem in a study conducted in China from 2017 to 2019 [16]. In Ghana, however, 64% of the *E. coli* from cattle demonstrated multidrug resistance, although none showed resistance to amikacin, which contrasts with our findings. However, all the Ghanaian isolates were resistant to meropenem. Among the 24 different resistance profiles obtained, most isolates were resistant to ampicillin (55%), followed by tetracycline (26%), sulfamethoxazole + trimethoprim (9.5%), chloramphenicol (7%), gentamicin and cefotaxime (5%), and ciprofloxacin (2%) [19]. 

This study analyzed the antibiotic-resistant *E. coli* profile between calves and adult cattle, highlighting a high level of *E. coli* resistance in calf feces from dairy and beef breeds such as Barrosã, Cachena, and Minhota. Calves are considered as reservoirs for antimicrobial-resistant *E. coli* [20], and they may pose a risk to human health through meat consumption, suggesting the acquisition of antibiotic resistance very early in their lives. In our study, 39% of the fecal *E. coli* were identified as ESBL producers; however, when considering only isolates from Holstein-Friesian cattle, this proportion increased to 59% (Figure S1). Similarly, another study found that 47.5% of fecal ESBL-producing *E. coli* in dairy cattle showed that calves have a higher likelihood of carrying ESBL-producing *E. coli* compared to adult cows [15]. An ESBL phenotype was identified in 21% of the *E. coli* obtained from calves [20]. In contrast, a different study found that only 1 of 120 cattle fecal *E. coli* isolates was identified as an ESBL producer [21]. *Escherichia coli* strains found in neonatal calf diarrhea often exhibit resistance to multiple antibiotics commonly used in the beef industry, including beta-lactams and tetracyclines [22,23]. The European Food Safety Authority (EFSA) recommends avoiding feeding calves with colostrum and milk containing antimicrobial residues that may promote resistance, especially to critically important antimicrobials. In dairy cattle, two key factors may contribute to the exposure of calves to antibiotics or their residues: (i) after calving, calves are fed colostrum from cows that may have been treated with antibiotics, and (ii) during lactation, calves are fed waste milk (milk from cows treated with antimicrobials, which is prohibited from sale) to minimize economic losses [24]. Jarrige et al. [20] reported an association between calves treated with antimicrobials or fed milk from treated cows and increased *E. coli* resistance to amoxicillin, gentamicin, tetracycline and trimethoprim + sulfonamide. However, no association was shown between antimicrobial resistance and consumption of colostrum by calves from cows treated with antimicrobials at dry-off. Consistent with previous studies, antimicrobial use and the prevalence of antimicrobial-resistant bacteria are typically high in calf fattening, with antimicrobial-resistant genes spreading clonally among calves. Notably, the median number of antimicrobial-resistant genes conferring resistance to specific antimicrobials tends to decrease by the end of the rearing period [25]. In our case, calves from all the breeds, especially dairy cattle, showed higher levels of antibiotic-resistant *E. coli* compared to adults. These results align with previous findings, showing a discrepancy in the phenotypic resistance level of isolates between calves and adult cattle, with higher resistance observed in calves than in cow isolates [26]. Moreover, calves fed with waste milk exhibited higher levels of antibiotic-resistant *E. coli* compared to those fed with tank milk, specifically showing resistance to tetracyclines (63%), ampicillin (38%), gentamycin (13%), and sulfamethoxazole/trimethoprim (13%) [26]. These findings indicate that exposing young animals to antibiotics may promote the development of antimicrobial resistance in their gut microbiota, including in bacteria like *E. coli* [26]. 

Our study identified seven antibiotic resistance genes belonging to five classes of antibiotics in *E. coli* isolates from Holstein-Friesian cattle, in contrast to isolates from native cattle breeds. The most prevalent genes included *sul*2 (69%, sulfamethoxazole), *bla*_CTX-M_ (45%, beta-lactams), *aac*(3′)-IV (41%, aminoglycosides), and *aac*(6′)-Ib-cr (31%, fluoroquinolones). A previous study also reported the prevalence of *gyrA* (74%, quinolones), *tetB* (70%), *sul2* (67%), and *bla_TEM_* (56%) as the most prevalent genes in *E. coli* isolates from the manure and feces of dairy cattle [16]. In our study (Table S1), the comparison between the phenotypic and genotypic detection of antibiotic resistance was noted to be variable. *Escherichia coli* isolates carrying antibiotic resistance genes were more prevalent than those exhibiting phenotypic resistance to beta-lactams, sulfonamides, and aminoglycosides. The opposite was observed for tetracycline and quinolone-resistant isolates [16]. In another study conducted in Ghana, analyzing β-lactamase genes, only *bla*_CTX-M_ was detected in one isolate, while the *bla*_SHV_ and *bla*_TEM_ genes were not found [20]. Conversely, the *bla*_CTX-M -1/15_ and *bla*_TEM_ genes were identified in all the bovine *E. coli* isolates from farms in Greece [27].

*Escherichia coli* isolates from native breed cows showed a lower variety of antibiotic resistance genes compared to calves. For instance, one of the two isolates from Minhota calves (farm 7) exhibited an MDR profile (Table S2), even though no antibiotics were administered in the three months prior to sampling. Another study reported that isolates from calves (60%) fed with tank milk showed higher antibiotic resistance than those from cows (16%). The resistance was predominantly to aminoglycosides (63%), sulfonamides (63%, *sul*1, *sul*2, *sul*3), tetracyclines (63%, *tet*A, *tet*B) and beta-lactams (38%, *bla*_TEM-1b_, *bla*_TEM-57_, *bla*_CTX-M1_). This finding confirms that antibiotic resistance genes decrease as animals age [26]. 

The discrepancies in antibiotic-resistant bacteria or genes among distinct breeds, as obtained in various studies, may be related to the varying levels of antibiotic use in these animals, exerting direct pressure on their commensal bacteria. Nonetheless, this study confirms the presence of antibiotic-resistant *E. coli* in all the analyzed breeds, including native national breeds. These animals can serve as potential reservoirs for the dissemination of these bacteria and resistance genes to other bacteria across different ecosystems. 

The detection of antibiotic resistance genes, such as *sul2* and *bla*_CTX-M1_, highlights bacterial resistance to antibiotics used in both veterinary and human medicine. The presence of these resistant bacteria in food-producing animals or their by-products underscores the significance of these findings within the One Health framework. Monitoring the transmission and spread of antibiotic-resistant bacteria is essential due to the interconnectedness of human, animal, and environmental health.

## 4. Materials and Methods

### 4.1. Sample Size and Geographical Distribution

**Figure 5:**
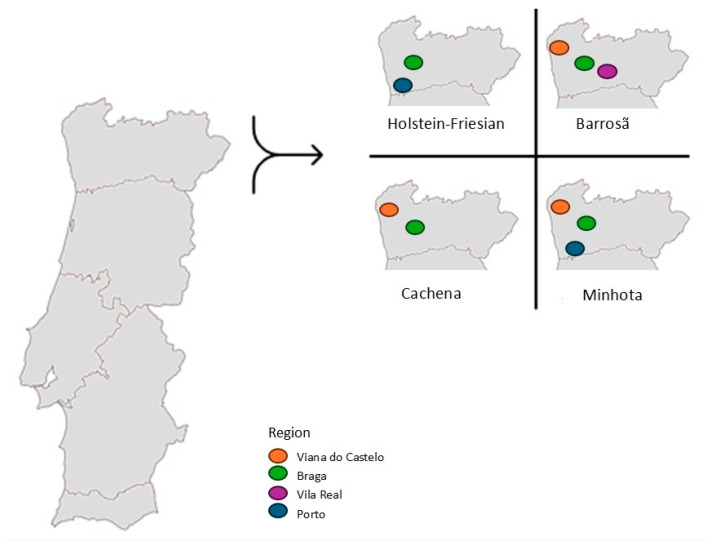
Spatial distribution of the bovine farms based on the four breeds included in this study. Each circle represents a sample collection region.

A total of 640 individual bovine fecal swab samples were collected from 40 different farms in Northern Portugal between February and June 2023. This included 10 intensive dairy farms with Holstein-Friesian cattle (an average of 186 animals per farm) and 30 extensive farms raising three native Portuguese breeds: Barrosã (an average of 43 animals per farm), Cachena (an average of 55 animals per farm), and Minhota (an average of 70 animals per farm) (Table 2, Figure 5). Briefly, 160 fecal samples from the 10 farms were collected per breed, corresponding to 16 samples per farm, which were pooled based on the age group (8 calves and 8 cows per farm). A total of 640 samples were grouped into 80 pools: 40 pools corresponded to calves, each consisting of 8 individual fecal samples, and 40 pools corresponded to adults, each also consisting of 8 individual fecal samples. Each pool was composed of 1 mL from each of the 8 individual fecal samples, thoroughly homogenized. Information regarding the farm’s location, total number of animals per farm, productive aptitude and production system, origin of colostrum/milk given to calves, and history of antibiotic administration within the last three months before sampling is presented in Table 2.

The intensive production of dairy farming, exemplified by the Holstein-Friesian breed, is characterized by a high number of animals per farm, with a focus on maximizing milk production through intensive management practices. The animals are kept in confinement, usually in barns or enclosed facilities, and are fed highly nutritious and controlled diets to ensure consistent and high milk production throughout the year. In addition, calves are fed their mother’s milk but can also be given discarded milk (milk that is unsuitable for human consumption or sale) and replacement milk (commercial milk).

In contrast, all the native bovines were listed in the genealogical register of their respective breeds. This conventional small-scale production system is characterized by extensive and transhumant production methods. In the extensive system, cattle spend part of their time in pastures but also receive supplementary feed, such as grains or silage, to ensure adequate nutrition, especially during drought periods or winter when the pasture availability is reduced. The transhumant system is a cattle-raising method where animals are seasonally moved in search of better pastures. This movement occurs between different areas throughout the year, depending on the climatic seasons and the availability of food. Both systems are adapted to the environment, balancing production costs and herd productivity. These animals are utilized for the production of food, raw materials and labor in rural agriculture. The calves are primarily fed their own mother’s milk and/or milk from other cows on the same farm (Table 2).

### 4.2. Bacterial Collection and Species Identification

One milliliter of the collected and pooled samples was inoculated in buffered peptone water (BPW) (pre-enriched in non-selective liquid medium, Scharlau^®^), allowing all the bacteria in the samples to grow freely without restricting or promoting the growth of any specific bacterial group. Simultaneously, the samples were also inoculated in BPW supplemented with 4 μg/mL of cefotaxime. Following this, a loop was used to inoculate onto MacConkey agar (Liofilchem, Roseto degli Abruzzi, Italy), either without antibiotics or supplemented with the previously mentioned cefotaxime (4 μg/mL), and onto Chromogenic Coliform Agar (Oxoid, Hants, UK). Isolates exhibiting typical *E. coli* morphology were selected and identified by MALDI-TOF MS. These isolates were stored in Trypticase Soy Broth with 15% glycerol at −80 °C. 

### 4.3. Antimicrobial Susceptibility Testing and ESBL Activity

Antibiotic susceptibility testing (AST) was performed by the disk diffusion method in Mueller-Hinton II agar, against 10 different antibiotics: ampicillin (10 μg); amoxicillin + clavulanic acid (20 μg + 10 μg); cefotaxime (5 μg); imipenem (10 μg); aztreonam (30 μg); gentamicin (10 μg); amikacin (30 μg); tetracycline (30 μg); trimethoprim + sulfamethoxazole (1.25 + 23.75 μg); and ciprofloxacin (5 μg). This selection includes antibiotics used in both veterinary and human medicine, as well as those exclusive to human medicine, reflecting the dual concern of addressing antibiotic use across sectors while recognizing the critical roles these drugs play in both public health and veterinary medicine. *Escherichia coli* ATCC 25922 was used as a control strain. The classification of isolates as susceptible or resistant was based on the corresponding breakpoints for each antibiotic. The results were interpreted according to the EUCAST guidelines [28], and for cefotaxime and tetracycline, the interpretation followed the CLSI guidelines [29]. The extended-spectrum β-lactamase (ESBL) production phenotype was assessed using the double-disk synergy test, which involved ampicillin, cefotaxime, amoxicillin + clavulanic acid, and aztreonam discs. The interpretations were conducted according to the EUCAST guidelines [28].

### 4.4. Analysis of Antibiotic Resistance Genes 

Representative *E. coli* isolates were selected based on the breed of origin and their phenotypic antibiotic resistance profiles. DNA was extracted using the boiling method, as previously described [30]. To detect antimicrobial resistance genes, PCR assays were performed with the Xpert Fast Hotstart Mastermix (GRiSP Research Solutions, Porto, Portugal) following the manufacturer’s instructions. The assays targeted some of the most frequent genes encoding resistance to beta-lactams (*bla*_TEM_, *bla*_SHV_, and *bla*_CTX-M_), tetracyclines (*tet*B), sulfamethoxazole (*sul*2), fluoroquinolones (*aac*(6′)-Ib-cr) and aminoglycosides (*aac*(3′)-IV), as previously described [11,31,32,33,34,35,36]. The PCR conditions followed the manufacturer’s instructions for the Xpert Fast Hotstart Mastermix, with the annealing temperatures adjusted for each primer pair (Table S2). Positive controls were used in all the PCR assays from the strain collection, kindly provided by the CCP—Culture Collection of Porto. The nucleotide sequence was amplified using a thermocycler (MJ Mini, Bio-Rad, Hercules, CA, USA). The PCR assay results were analyzed by electrophoresis containing GelRed fluorescent dye (GelRed^®^ nucleic acid stain, Biotium, Fremont, CA, USA). A molecular weight marker (NzyDNA Ladder V, Nzytech, Lisbon, Portugal) was included to aid interpretation of the results, which were visualized on a transilluminator (ChemiDoc, Bio-Rad, Hercules, CA, USA).

## 5. Conclusions

This study provides evidence of multidrug-resistant *E. coli* carrying antibiotic resistance genes in all four breeds examined. Strains from the Holstein-Friesian samples exhibited more extensive resistance profiles, likely linked to historical antibiotic use on these farms. In contrast, native cattle from less-explored ecological niches, where antibiotics had not been for at least three months prior to sampling, showed lower resistance levels. These findings underscore the risk to food safety and public health posed by antibiotic-resistant bacteria in production animals, regardless of whether they are raised in intensive or extensive systems.

Notably, *E. coli* isolates from both calf and adult dairy and beef cattle demonstrated high resistance levels, highlighting the potential for resistant bacteria to spread to humans through environmental pathways or direct animal contact. Cattle may serve as significant reservoirs of antibiotic-resistant bacteria and resistance genes, with direct implications for global health.

Addressing these challenges requires public education and targeted training for healthcare professionals and farmers to reduce the misuse and overuse of antibiotics. Systematic monitoring of antimicrobial use and resistance is critical for identifying high-risk species, regions, and exposure pathways that drive resistance development.

Finally, resistance to human-specific antibiotics, such as aztreonam, resistance to critically important antibiotics like gentamicin, and resistance to highest priority critically important antimicrobials, such as ciprofloxacin, as observed in isolates from both dairy cattle and native breeds, emphasizes the urgency of implementing national and global standards for prudent antimicrobial use in animals. This approach aligns with the WHO’s recommendations and reinforces the importance of integrated One Health strategies to combat antibiotic resistance on a global scale. 

## Figures and Tables

**Table 1:** General genotypic characterization of the antimicrobial resistance profile of *E. coli* by age group (n = 22 adults, n = 27 calves) isolated from all the cattle breeds used in this study.

Gene	Adults		Calves		Total	
	No.	%	No.	%	No.	%
*bla*_TEM_	5	23	7	26	12	24
*bla*_SHV_	2	9	3	11	5	10
*bla*_CTX-M_	10	45	12	44	22	45
*tet*B	5	23	8	30	13	27
*sul*2	16	73	18	67	34	69
*aac*(3′)-IV	10	45	10	37	20	41
*aac*(6′)-Ib-cr	8	36	7	26	15	31

No.—number of isolates. %—percentage.

**Table 2:** General characteristics of the bovine farms (dairy and autochthones Portuguese breeds) included in this study.

Breed/Farm	Region	Total Number of Animals	Antibiotic Administration in the Last 3 Months *	Productive Aptitude	Origin of Milk Given to Calves
Holstein-Friesian					
1	Braga	170	Yes	Milk	Cow, Discard
2	Braga	150	Yes	Milk	Discard, Replacement
3	Porto	342	Yes	Milk	Cow, Discard, Replacement
4	Braga	140	Yes	Milk	Cow
5	Porto	140	Yes	Milk	Discard, Replacement
6	Braga	320	Yes	Milk	Discard, Replacement
7	Porto	140	Yes	Milk	Discard, Replacement
8	Porto	150	Yes	Milk	Cow, Discard
9	Porto	215	Yes	Milk	Cow, Replacement
10	Porto	98	Yes	Milk	Cow, Discard
Barrosã					
1	Vila Real	59	No	Meat	Cow
2	Vila Real	34	No	Meat	Cow
3	Vila Real	47	No	Meat	Cow
4	Vila Real	28	No	Meat	Cow
5	Vila Real	86	No	Meat	Cow
6	Viana do Castelo	38	No	Meat	Cow
7	Vila Real	31	No	Meat	Cow
8	Braga	51	No	Meat	Cow
9	Viana do Castelo	20	No	Meat	Cow
10	Viana do Castelo	40	No	Meat	Cow
Cachena					
1	Viana do Castelo	104	No	Meat	Cow
2	Viana do Castelo	51	No	Meat	Cow
3	Viana do Castelo	65	No	Meat	Cow
4	Viana do Castelo	45	No	Meat	Cow
5	Braga	27	No	Meat	Cow
6	Viana do Castelo	72	No	Meat	Cow
7	Viana do Castelo	64	No	Meat	Cow
8	Viana do Castelo	42	No	Meat	Cow
9	Braga	55	No	Meat	Cow
10	Viana do Castelo	27	No	Meat	Cow
Minhota					
1	Braga	38	No	Meat	Cow
2	Braga	64	No	Meat	Cow
3	Braga	68	No	Meat	Cow
4	Viana do Castelo	173	No	Meat	Cow
5	Braga	81	No	Meat	Cow
6	Braga	22	Yes	Meat	Cow
7	Braga	115	No	Meat	Cow
8	Braga	76	Yes	Meat	Cow
9	Braga	22	No	Meat	Cow
10	Porto	43	No	Meat	Cow

* Holstein-Friesian breed: amoxicillin, penicillin, colistin sulfate and streptomycin. Minhota breed: oxytetracycline.

## Data Availability

Data are contained within the article and supplementary materials.

## Supplementary Materials

The following supporting information can be downloaded at https://www.mdpi.com/article/10.3390/antibiotics13121208/s1, Table S1: Phenotypic and genotypic characteristics of *E. coli* isolated from cattle breeds. Table S2: Nucleotide sequences, with the annealing temperature (AT) and expected amplicon size, used in this study. Figure S1. Phenotypic characterization of antimicrobial resistance in *E. coli* by production system. Figure S2. Screening of antibiotic-resistant genes amplified in this study.
