# Supplementary material for: Dairy Cattle and the Iconic Autochthonous Cattle in Northern Portugal Are Reservoirs of Multidrug-Resistant Escherichia coli
Source: Antibiotics (Basel). 2024 Dec 11;13(12):1208. doi: 10.3390/antibiotics13121208 (PMC11672626; doi:10.3390/antibiotics13121208)
Supplement: Supplementary file 1 [file antibiotics-13-01208-s001.zip › antibiotics-3321471-supplementary.pdf]

antibiotics

Supplementary Materials

Dairy Cattle and the Iconic Autochthonous Cattle in Northern Portugal Are Reservoirs of Multidrug-Resistant *Escherichia coli*

Sandra Quinteira [1,2,3,4], Rui Dantas [4,5], Luís Pinho [6], Carla Campos [7,8], Ana R. Freitas [4,9,10], Nuno V. Brito [4,11] and Carla Miranda [4,12,*]

[1] CIBIO—Research Center in Biodiversity and Genetic Resources, InBIO, Research Network in Biodiversity and Evolutionary Biology, Associated Laboratory, University of Porto, Campus de Vairão, Rua Padre Armando Quintas 7,4485-661 Vairão, Portugal; sandra.quinteira@ipsn.cespu.pt
[2] BIOPOLIS Program in Genomics, Biodiversity and Land Planning, Campus de Vairão, Rua Padre Armando Quintas 7, 4485-661 Vairão, Portugal
[3] Department of Biology, Faculty of Sciences, University of Porto, Rua do Campo Alegre s/n, 4169-007 Porto, Portugal
[4] UCIBIO—Applied Molecular Biosciences Unit, University Institute of Health Sciences (1H-TOXRUN, IUCS-CESPU), Avenida Central de Gandra 1317, 4585-116 Paredes, Portugal; rui.dantas@iucs.cespu.pt (R.D.); ana.freitas@iucs.cespu.pt (A.R.F.); nuno.brito@iucs.cespu.pt (N.V.B.)
[5] ACRC—Associação Criadores da Raça Cachena, Parque Empresarial de Paçô, Rua da Roca 107, 4970-249 Arcos de Valdevez, Portugal
[6] Department of Veterinary Clinics, Abel Salazar Biomedical Sciences Institute, University of Porto, Rua Jorge de Viterbo Ferreira 228, 4050-313 Porto, Portugal; lapinho@icbas.up.pt
[7] Instituto Português de Oncologia do Porto Francisco Gentil, Rua Dr. António Bernardino de Almeida, 4200-072 Porto, Portugal; carla.campos@ipoporto.min-saude.pt
[8] Escola Superior de Saúde, Instituto Politécnico do Porto, Rua Dr. António Bernardino de Almeida, 4200-072 Porto, Portugal
[9] UCIBIO—Applied Molecular Biosciences Unit, Faculty of Pharmacy, University of Porto, Rua Jorge de Viterbo Ferreira 228, 4050-313 Porto, Portugal
[10] Associate Laboratory i4HB, Institute for Health and Bioeconomy, Faculty of Pharmacy, University of Porto, Rua Jorge de Viterbo Ferreira 228, 4050-313 Porto, Portugal
[11] CISAS—Center for Research and Development in Agrifood Systems and Sustainability, Higher Agricultural School, Polytechnic Institute of Viana do Castelo, Rua Escola Industrial e Comercial de Nun'Álvares, 4900-347 Viana do Castelo, Portugal
[12] LAQV-REQUIMTE—Associated Laboratory for Green Chemistry of the Network of Chemistry and Technology, University NOVA of Lisbon, Campus da Caparica, 1099-085 Caparica, Portugal
* Correspondence: carla.miranda@iucs.cespu.pt

Citation: Quinteira, S.; Dantas, R.; Pinho, L.; Campos, C.; Freitas, A.R.; V. Brito, N.; Miranda, C. Dairy Cattle and the Iconic Autochthonous Cattle in Northern Portugal Are Reservoirs of Multidrug-Resistant *Escherichia coli*. *Antibiotics* **2024**, *13*, 1208. https://doi.org/10.3390/antibiotics13121208

Academic Editors: Marc Maresca, Manuel Simões and Carlos M. Franco

Received: 2 November 2024
Revised: 7 December 2024
Accepted: 10 December 2024
Published: 11 December 2024

Copyright: © 2024 by the authors. Licensee MDPI, Basel, Switzerland. This article is an open access article distributed under the terms and conditions of the Creative Commons Attribution (CC BY) license (https://creativecommons.org/licenses/by/4.0/).

Table S1: Phenotypic and genotypic characteristics of *E. coli* isolated from cattle breeds.

No. Isolate	Animal age	No. Farm	Breed	Antibiotic resistance phenotype*	MDR	Antibiotic resistance genotype
1	Calf	1	H-Friesian	AMP, AMC, CTX, ATM, CIP, CN, AK, TE	Yes	bla_{CTX}, *tetB*, *sul2*, *aac (3′) IV*, *aac(6′)-ib-cr*
2	Adult	1	H-Friesian	AMP, AMC, CTX, ATM, CN	No	bla_{TEM}, bla_{CTX}, *tetB*, *sul2*, *aac (3′) IV*
3	Adult	2	H-Friesian	AK	No	*tetB*, *sul2*
4	Adult	2	H-Friesian	AMP, CN	No	*aac (3′) IV*
5	Calf	2	H-Friesian	AMP, AK, TE	Yes	bla_{TEM}, bla_{CTX}, *aac (3′) IV*
6	Calf	4	H-Friesian	AMP, AMC, TE	No	*tetB*, *sul2*
7	Calf	4	H-Friesian	AMP, AMC, CTX, ATM, TE, STX	Yes	*tetB*, *sul2*
8	Adult	4	H-Friesian	AMP, AMC, CN, TE	Yes	*tetB*
9	Calf	5	H-Friesian	AMP, AMC, CTX, ATM, CIP, CN, TE, STX	Yes	bla_{CTX}, *sul2*
10	Adult	5	H-Friesian	AMP, AMC, CTX, ATM, CIP, CN, AK, TE, STX	Yes	bla_{CTX}, *tetB*, *sul2*
11	Adult	5	H-Friesian	AMP, AMC, CTX, ATM, CIP, CN, TE, STX	Yes	bla_{CTX}, *sul2*, *aac (3′) IV*, *aac(6′)-ib-cr*
12	Calf	6	H-Friesian	CIP, CN, AK, STX	Yes	*sul2*
13	Calf	6	H-Friesian	AMP, AMC, CTX, CIP, CN, TE, STX	Yes	bla_{SHV}, *tetB*, *sul2*, *aac (3′) IV*
14	Calf	6	H-Friesian	AMP, AMC, CN, TE, STX	Yes	bla_{TEM}, *tetB*, *sul2*
15	Calf	7	H-Friesian	AMP, CTX, ATM, CN, TE, STX	Yes	bla_{CTX}, *sul2*, *aac (3′) IV*, *aac(6′)-ib-cr*
16	Adult	7	H-Friesian	AMP, CTX, ATM, CN, AK, STX	Yes	bla_{CTX}, *sul2*, *aac (3′) IV*, *aac(6′)-ib-cr*
17	Adult	7	H-Friesian	AMP, CTX, ATM, CN, TE, STX	Yes	-
18	Calf	8	H-Friesian	AMP, CTX, ATM, STX	No	bla_{CTX}, *sul2*
19	Calf	8	H-Friesian	CIP	No	*sul2*
20	Adult	8	H-Friesian	AMP, AMC, CTX, ATM, STX	No	bla_{TEM}, bla_{CTX}, *tetB*, *sul2*, *aac (3′) IV*, *aac(6′)-ib-cr*
21	Calf	9	H-Friesian	AMP, AMC, TE, STX	Yes	-
22	Calf	9	H-Friesian	AMP, AMC, CTX, ATM, CIP, CN, TE, STX	Yes	bla_{TEM}, bla_{CTX}, *sul2*, *aac (3′) IV*, *aac(6′)-ib-cr*
23	Calf	9	H-Friesian	AMP, CN, TE, STX	Yes	-
24	Adult	9	H-Friesian	AMP, AMC, CTX, ATM, CN, TE, STX	Yes	bla_{CTX}, *sul2*, *aac (3′) IV*
25	Adult	9	H-Friesian	AMP, AMC, CTX, ATM, CN, TE, STX	Yes	bla_{CTX}, *sul2*, *aac (3′) IV*, *aac(6′)-ib-cr*
26	Calf	10	H-Friesian	AMP, AMC, CTX, ATM, CIP, CN, TE, STX	Yes	bla_{TEM}, bla_{SHV}, bla_{CTX}, *sul2*, *aac (3′) IV*, *aac(6′)-ib-cr*
27	Calf	10	H-Friesian	AMP, CTX, ATM, CN, TE, STX	Yes	bla_{TEM}, bla_{CTX}, *tetB*, *sul2*, *aac (3′) IV*, *aac(6′)-ib-cr*
28	Adult	10	H-Friesian	AMP, AMC, CTX, CN, TE	Yes	bla_{TEM}, bla_{CTX}, *sul2*, *aac (3′) IV*, *aac(6′)-ib-cr*
29	Adult	10	H-Friesian	AMP, AMC, CTX, ATM, CN, TE, STX	Yes	bla_{TEM}, bla_{SHV}, bla_{CTX}, *sul2*, *aac (3′) IV*, *aac(6′)-ib-cr*
30	Adult	1	Barrosã	AMP, AMC, CN, AK, TE, CIP, STX	Yes	*sul2*
31	Adult	2	Barrosã	-	No	-
32	Calf	3	Barrosã	AMP, CN, TE	Yes	*tetB*, *sul2*
33	Calf	5	Barrosã	AMP, CN, AK, TE, CIP	Yes	*tetB*, *aac (3′) IV*
34	Calf	5	Barrosã	AMP, TE, STX	Yes	-
35	Calf	6	Barrosã	-	No	-

36	Adult	6	Barrosã	AK	No	bla_{SHV}, *sul2*
37	Adult	6	Barrosã	AK	No	-
38	Adult	7	Barrosã	CN	No	-
39	Calf	7	Barrosã	-	No	-
40	Adult	8	Barrosã	AMP, TE, STX	Yes	bla_{TEM}, *sul2, aac (3') IV, aac(6')-ib-cr*
41	Calf	8	Barrosã	STX	No	bla_{CTX}, *sul2, aac (3') IV*
42	Calf	1	Cachena	AMP, CTX, TE, SXT	Yes	bla_{TEM}, bla_{CTX}, *sul2, aac(3') IV*
43	Adult	5	Cachena	-	No	*sul2, aac(6')-ib-cr*
44	Adult	5	Cachena	AK	No	*sul2*
45	Calf	5	Cachena	AK	No	-
46	Calf	10	Cachena	AK	No	-
47	Calf	1	Minhota	-	No	bla_{CTX}, *sul2, aac(6')-ib-cr*
48	Adult	7	Minhota	AMC, CTX, ATM, AK	No	bla_{CTX}, *sul2*
49	Calf	7	Minhota	AMC, CN, TE, SXT	Yes	bla_{TEM}, bla_{SHV}, bla_{CTX}, *sul2, aac(6')-ib-cr*

(No.: number; MDR: multidrug resistance; AMP: ampicillin; AMC: amoxicillin + clavulanic acid; CTX: cefotaxime; ATM: aztreonam; IPM: imipenem; CIP: ciprofloxacin; CN: gentamicin; AK: amikacin; TE: tetracycline; SXT: trimethoprim + sulfamethoxazole)

Table S2: Nucleotide sequences with annealing temperature (AT) and expected amplicon size, used in this study.

Primer	Nucleotide sequence (5′ to 3′)	Size (bp)	AT (°C)	Reference
blaCTX-M	ATGTGCAGYACCAGTAARGT<br>TGGGTRAARTARGTSACCAGA	593	50	Lim et al. [31]
blaSHV	GGTTATGCGTTATATTCGCC<br>TTAGCGTTGCCAGTGCTC	867	60	Lim et al. [31]
blaTEM	GAGTATTCAACATTTTCGT<br>ACCAATGCTTAATCAGTGA	857	50	Maynard et al. [33]
tetB	CTCAGTATTCCAAGCCTTTG<br>CTAGCACTTGTCTCCTGTT	416	57	Guardabassi et al. [34]
sul2	CGGCATCGTCAACATAACCT<br>TGTGCGGATGAAGTCAGCTC	721	55	Maynard et al. [33]
aac(3′)-IV	CTTCAGGATGGCAAGTTGGT<br>TCATCTCGTTCTCCGCTCAT	286	60	Sáenz et al. [35]
aac(6′)-Ib-cr	CGGCATCGTCAACATAACCT<br>TGTGCGGATGAAGTCAGCTC	482	55	Park et al. [36]

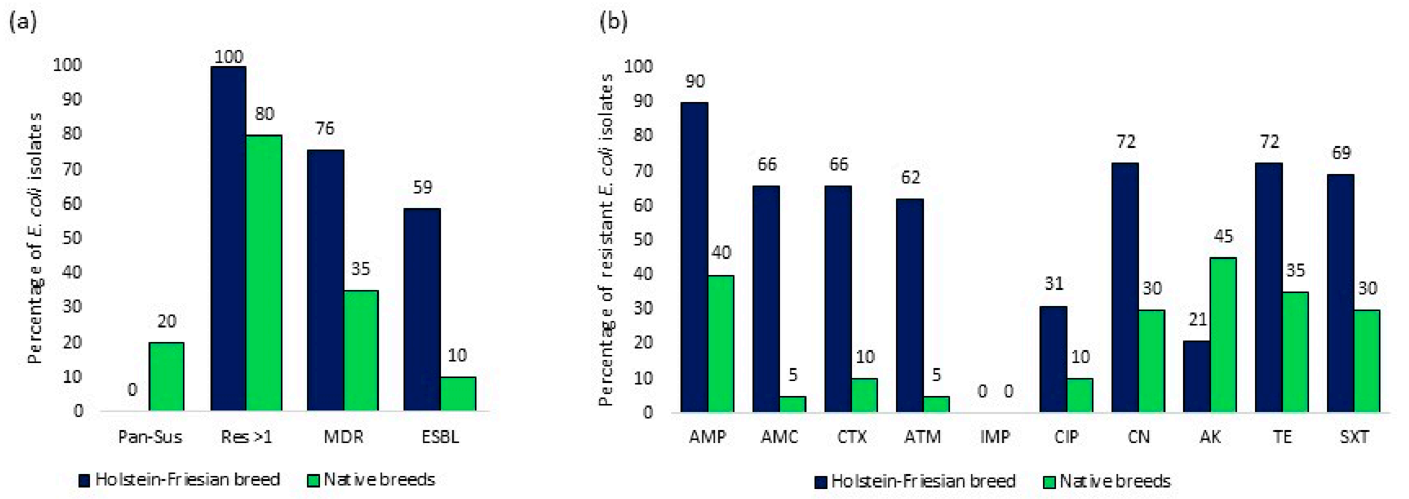

Figure S1. Phenotypic characterization of antimicrobial resistance in *E. coli* by production system: intensive system (including the Holstein-Friesian breed, n=29) and extensive system (including the Barrosã, Cachena, and Minhota breeds, n=20). (**a**) Antimicrobial susceptibility profiles; (**b**) Antimicrobial resistance of *E. coli* isolates. (Pan-sus- pan-susceptibility; Res ≥1- resistance to ≥1 antibiotic; MDR- Multidrug-resistant phenotype; ESBL- Extended-spectrum β-lactamase production phenotype; AMP: ampicillin; AMC: amoxicillin + clavulanic acid; CTX: cefotaxime; ATM: aztreonam; IPM: imipenem; CIP: ciprofloxacin; CN: gentamicin; AK: amikacin; TE: tetracycline; SXT: trimethoprim + sulfamethoxazole)

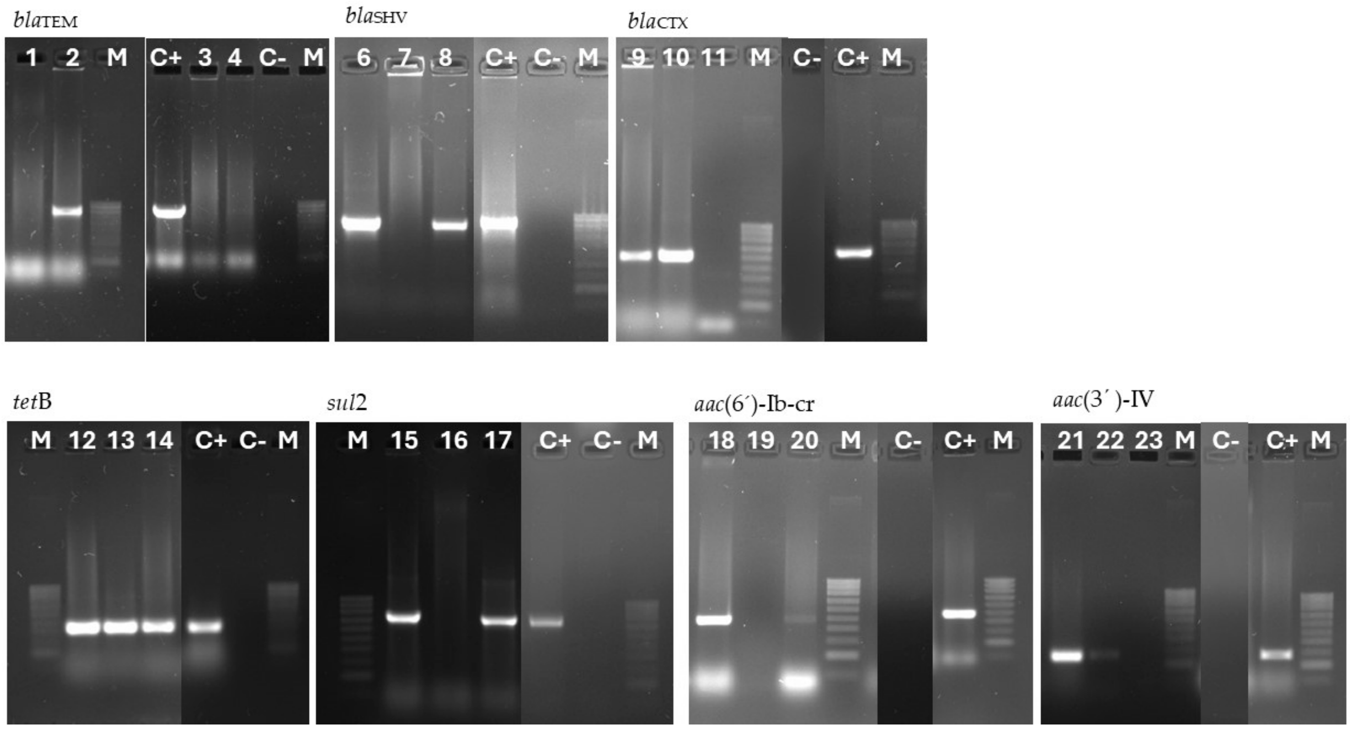

Figure S2. Screening of antibiotic-resistant genes amplified in this study. 1-23: isolates. M: molecular weight marker (100-1000 bp). C+: positive control. C-: negative control.
